# Cucurbitaceae Seed Protein Hydrolysates as a Potential Source of Bioactive Peptides with Functional Properties

**DOI:** 10.1155/2017/2121878

**Published:** 2017-10-17

**Authors:** César Ozuna, Ma. Fabiola León-Galván

**Affiliations:** Departamento de Alimentos, Posgrado en Biociencias, División de Ciencias de la Vida, Universidad de Guanajuato, Campus Irapuato-Salamanca, Carretera Irapuato-Silao Km 9, 36500 Irapuato, GTO, Mexico

## Abstract

Seeds from Cucurbitaceae plants (squashes, pumpkins, melons, etc.) have been used both as protein-rich food ingredients and nutraceutical agents by many indigenous cultures for millennia. However, relatively little is known about the bioactive components (e.g., peptides) of the Cucurbitaceae seed proteins (CSP) and their specific effects on human health. Therefore, this paper aims to provide a comprehensive review of latest research on bioactive and functional properties of CSP isolates and hydrolysates. Enzymatic hydrolysis can introduce a series of changes to the CSP structure and improve its bioactive and functional properties, including the enhanced protein solubility over a wide range of pH values. Small-sized peptides in CSP hydrolysates seem to enhance their bioactive properties but adversely affect their functional properties. Therefore, medium degrees of hydrolysis seem to benefit the overall improvement of bioactive and functional properties of CSP hydrolysates. Among the reported bioactive properties of CSP isolates and hydrolysates, their antioxidant, antihypertensive, and antihyperglycaemic activities stand out. Therefore, they could potentially substitute synthetic antioxidants and drugs which might have adverse secondary effects on human health. CSP isolates and hydrolysates could also be implemented as functional food ingredients, thanks to their favorable amino acid composition and good emulsifying and foaming properties.

## 1. Introduction

**Figure 1:**
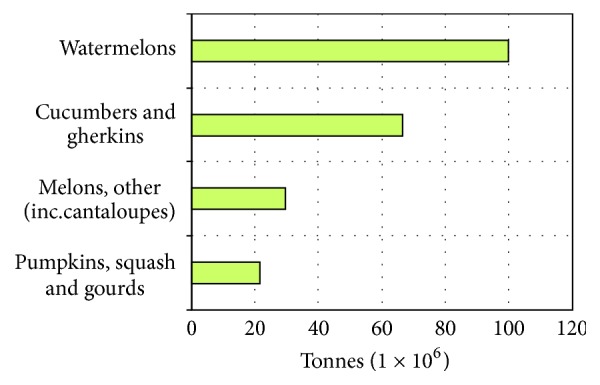
World production of Cucurbitaceae fruits in 2014 [3].

The Cucurbitaceae family is formed by about 130 genera and 800 species, including squashes, pumpkins, melons, and gourds [1, 2].  Figure 1 shows the total world production of Cucurbitaceae fruits in 2014 (217,714,974 tonnes) divided into four major groups [3]. Cucurbitaceae plants are cultivated in warmer regions of the world and many of their parts are typically used as food, especially the fruit, the flowers, and the seeds [4, 5]. However, Cucurbitaceae seeds have long been used in indigenous diets as popular medicine, thanks to their bioactive and nutraceutical properties [6, 7].

Industrially, Cucurbitaceae seeds are normally used for the extraction of edible and drying oils which comprise about a half of the seed's weight [8]. The main byproduct in the oil production is the oil cake which is rich in carbohydrates and has a very high content of protein (60–65% [w/w]; [9, 10]). However, this nutritive and potentially bioactive byproduct is usually discarded as waste or only used as animal feed. Since Cucurbitaceae seeds also seem to be a valuable source of good quality proteins in terms of their amino acid composition; they could potentially be used as functional food ingredients [11–13]. Moreover, enzymatic hydrolysis can improve the bioactive and functional properties of proteins [14–16]. Therefore, Cucurbitaceae seed protein hydrolysates could be not only an interesting alternative to known food supplements but also a potential source of new bioactive peptides.

In recent years, Cucurbitaceae seed proteins and their hydrolysates have gained considerable attention from researchers and the body of evidence for their bioactive and functional properties is quickly growing. Therefore, the main objective of this paper is to provide a comprehensive review of latest research conducted in the field of Cucurbitaceae seed protein isolation and its subsequent enzymatic hydrolysis in order to obtain bioactive peptides with enhanced functional properties. At the same time, this review aims to put into perspective the lack of knowledge about bioactive peptides recoverable from Cucurbitaceae seed protein hydrolysates and thus, it aims to point out the many opportunities this area may provide for future research.

## 2. Generation of Cucurbitaceae Seed Protein Hydrolysates

**Figure 2:**
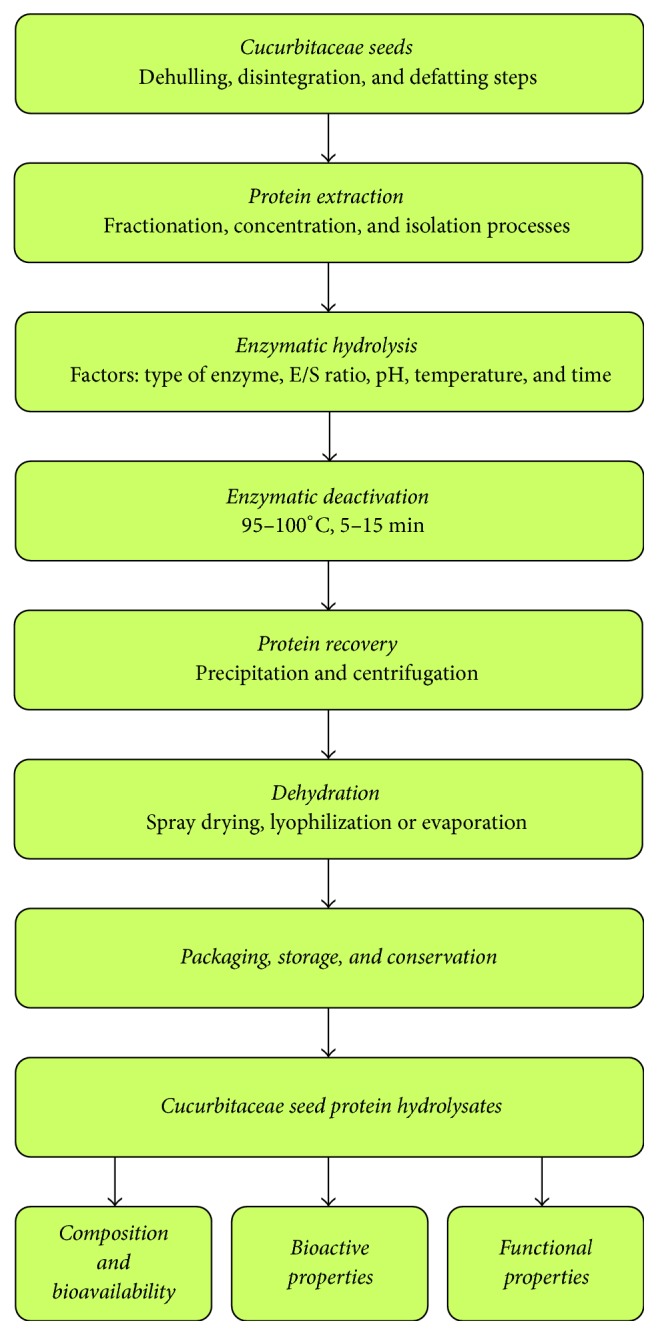
Generation of Cucurbitaceae seed protein hydrolysates.

This section describes the processes involved in the generation of Cucurbitaceae seed protein extracts and their enzymatic hydrolysates, starting with the extraction of the protein from the seeds themselves, then studying the main factors involved in the enzymatic hydrolysis of the Cucurbitaceae seed protein extracts and, finally, describing the steps involved in the obtainment of Cucurbitaceae seed protein hydrolysates (Figure 2).

### 2.1. Cucurbitaceae Seeds

First, Cucurbitaceae seeds are usually manually separated from the ripe fruit and they are dehulled if necessary and possible. In order to obtain the seed meal (sometimes referred to as seed flour), disintegrating and defatting steps are applied to the seeds. The disintegration is achieved by grinding or pulverizing the dehulled seeds. The defatting of the ground seeds is carried out with hexane and the resulting meal is dried at room temperature. The disintegration step may be repeated at this point. The resulting meal may also be passed through a 60-mesh sieve in order to obtain a fine powder [17, 18]. In the case of Cucurbitaceae seeds that are used in oil production (especially* Cucurbita pepo*, but also* Citrullus lanatus*), the residual oil cake resulting from the seed oil pressing is first ground and then defatted, following the same procedures as described above [10, 19]. The defatted Cucurbitaceae seed meal is then stored in a dry and cool place until used for the protein extraction.

### 2.2. Cucurbitaceae Seed Protein Extraction

At the laboratory scale, Cucurbitaceae seed protein extraction has been carried out mainly by means of fractionation, concentration, and isolation processes, resulting in potential food and pharmaceutical applications [9]. However, different factors (such as pH, temperature, ionic strength, solvent type, extraction time, and solid-liquid ratio, among others [19]) may affect the protein extraction process, thus making it rather complicated and unpractical to implement at industrial level.

Traditionally, the extraction of storage proteins from seeds has been carried out following Osborne [20] who classifies proteins according to their solubility in water (albumin), salt solutions (globulin), alkali solutions (glutelin), and alcohol solutions (prolamin). Nowadays, however, many Cucurbitaceae seed protein researchers deal directly with cucurbitin (11S globulin) which is the main storage protein in Cucurbitaceae seeds [10]. Cucurbitin is a hexameric globular protein whose subunits weigh 54 kDa each. In turn, each subunit consists of an acidic and a basic subunit which are disulfide-bonded and weigh 33 kDa and 22 kDa, respectively [21]. Peričin et al. [9] describe the standard procedure of cucurbitin extraction. First, the defatted seed meal is extracted with water and this fraction is discarded. Subsequently, the globulin fraction is extracted with NaCl solution at room temperature. The protein is then precipitated from the clarified extract by gradual addition of water. The precipitate is dissolved in standard buffer, the solution is clarified by centrifugation, and the cucurbitin is precipitated by addition of water [9, 10, 22].

On the other hand, high-yield protein isolates can be extracted from Cucurbitaceae seeds in alkaline conditions when not aiming only for cucurbitin but for all protein fractions in one [18, 19, 23–25]. First, the defatted seed meal is treated using NaOH solution (pH 8–10). Subsequently, the solution is centrifuged and an isoelectric precipitation of the protein from the supernatant is carried out, usually using dilute HCl (pH values about 4-5). The precipitate is then separated from the whey by centrifugation and the resulting protein pellet is dried in order to obtain the protein isolate. This process can be implemented to obtain protein isolates which consist of about 80–90% of protein. Moreover, Cucurbitaceae seed protein isolates have proven to have some bioactive and functional properties even before any hydrolysis takes place, so they make a very good comparison point for the Cucurbitaceae seed protein hydrolysates.

Following the extraction process, cucurbitin [10, 22], the protein isolate [23–27], or the defatted seed meal itself [17] can be hydrolyzed in order to obtain Cucurbitaceae seed protein hydrolysates. Generally, enzymatic hydrolysis is preferred for any type of protein since it can guarantee less drastic hydrolytic conditions and more specific cleavage sites in comparison to chemical hydrolysis [28, 29].

### 2.3. Enzymatic Hydrolysis of Cucurbitaceae Seed Protein Extracts

The main factors described to influence hydrolysis of Cucurbitaceae seed protein are the type of enzyme used, the enzyme/substrate (E/S) ratio, pH value, temperature and time of hydrolysis [14]. These factors influence not only the degree of hydrolysis (DH, defined as the proportion of cleaved peptide bonds in a protein hydrolysate [30]), but also the molecular weight distribution of the peptides conforming the hydrolysates [29, 31] and their bioactive and functional properties [32]. For the research papers revised in this section, Table 1 summarizes the main hydrolysis conditions as well as the DH, molecular weight distribution, and the bioactive and functional properties of the Cucurbitaceae seed protein hydrolysates.

One of the frequently studied factors that can influence hydrolysis is the type of enzyme used. Since different proteolytic enzymes produce compounds with different physicochemical and nutritional characteristics, it is important to identify most suitable enzymes for each substrate. Thus, enzymatic hydrolysis of the peptide linkage between amino acids can result in a mixture of peptides of various molecular sizes and free amino acids, depending on the type of enzyme used [29]. The proteases that are normally used in Cucurbitaceae seed protein hydrolysis come from animals (Pepsin and Trypsin) or fungi and bacteria (Alcalase, Neutrase, and Flavourzyme) and they can be classified as endoproteases (Alcalase, Pepsin, and Trypsin), exoproteases, and complex mixtures of endo- and exoproteases (Flavourzyme). On the one hand, endoproteases produce relatively large peptides by hydrolyzing the peptide bonds within protein molecules at random. Exoproteases, on the other hand, specialize in hydrolyzing the terminal peptide bonds. Thus, they produce free amino acids by removing them one by one from either the N- or C-terminal sites of the protein molecule [29, 40]. However, often a mixture of free amino acids, dipeptides, tripeptides, and other short peptides (<1.5 kDa) is desirable for use in special formulations (see Section 3). In these cases, a sequential use of endo- and exoproteases is applied during hydrolysis (the former facilitating the action of the latter) for a more complete and specific degradation of the protein [29].

The specificity of the enzymes used in Cucurbitaceae seed protein hydrolysis is thought to result in different DH and molecular weight distributions of the hydrolysates [17, 22, 23]. Venuste et al. [17] analyzed the influence of four different enzymes (Alcalase, Flavourzyme, Protamex, and Neutrase) on the hydrolysis process of pumpkin* (Cucurbita moschata)* seed protein meal. The DH achieved by Alcalase, Flavourzyme, Protamex, and Neutrase was 13.84%, 11.80%, 8.74%, and 4.12%, respectively. According to the authors, Protamex and Neutrase are not able to break the pumpkin protein peptide bond efficiently enough to achieve a DH comparable to those of Alcalase and Flavourzyme. As for molecular weight distribution, a larger proportion of low-molecular weight peptide fractions (1–0.18 kDa) was achieved in the hydrolysis of the protein meal by Acalase and Protamex (57.20% and 50.90%, resp.) compared to Neutrase and Flavourzyme (34.43% and 25.83%, resp.). Thus, Alcalase turned out to be the most suitable enzyme for this particular protein in order to achieve both the highest DH and the highest proportion of low-molecular weight peptides in the hydrolysate.

Vaštag et al. [22] studied the influence of the type of enzyme (Alcalase and Pepsin) on the hydrolysis process of cucurbitin obtained from pumpkin (*Cucurbita pepo* L. c. v. “Olinka”) seed oil cake. Under the same E/S ratio and time conditions (0.02 g/1 g and 60 min, resp.), Alcalase achieved a higher DH (26.9 ± 1.1%) in comparison to Pepsin (18.7 ± 1.2%). The authors suggest that, due to the specificity of the two enzymes, the same protein was hydrolyzed at different peptide bonds, resulting in a different composition of the hydrolysates. Similarly, Arise et al. [23] studied the influence of enzyme type (Pepsin, Trypsin, and Alcalase) on the hydrolysis process of watermelon (*Citrullus lanatus* L.) seed proteins. In this study, Trypsin produced a higher DH (26.26 ± 0.27%) than Pepsin and Alcalase (19.38 ± 0.86 and 13.16 ± 1.82%, resp.). This trend was ascribed to the affinity of Trypsin to cleave at sites with C-terminal end, which could generate more amino acids than peptides. However, neither Vaštag et al. [22] nor Arise et al. [23] reported molecular weight distribution of the peptides in the hydrolysates. This kind of information can be very valuable for the determination of the peptide bioavailability and the functional properties of the protein hydrolysates.

Some researchers have used a sequence of different enzymes in the hydrolysis of Cucurbitaceae seed proteins [25, 26, 34]. Siddeeg et al. [34] and Siddeeg et al. [25] obtained a high proportion of low-molecular weight peptide fractions (1–0.18 kDa) in the hydrolysates of seinat (*Cucumis melo* var.* tibish*) seed proteins prepared using a sequential treatment with Pepsin-Trypsin [34] and Trypsin-Pepsin [25]. Additionally, in order to study the effect of the sequential use of different enzymes on the DH in Cucurbitaceae seed protein hydrolysates, Vaštag et al. [26] compared the use of Alcalase, Flavourzyme, and their sequential use in the hydrolysis of protein isolate from pumpkin (*Cucurbita pepo* L. c. v. “Olinka”) oil cake. In addition, three different E/S ratios were used for both Alcalase and Flavourzyme (1/757, 1/385, and 1/250 [w/w]), with higher E/S ratios yielding higher DH for both enzymes. At the same E/S ratios, Alcalase produced hydrolysates with higher DH than Flavourzyme over the entire period of the hydrolysis which indicates a higher proteolytic activity of Alcalase towards this type of protein. The highest DH values were reached at the E/S ratio of 1/250 [w/w] and the hydrolysis time of 60 min, 53.23 ± 0.70% for Alcalase, and 37.17 ± 1.05% for Flavourzyme. As for the sequential use of both enzymes, this proved to be more efficient than using both enzymes separately. The maximum DH reached by using Alcalase alone did not increase with prolonged hydrolysis. However, the addition of Flavourzyme (E/S 1/385 [w/w]) at 60 min increased the maximum DH to 69.29 ± 0.9% (at 120 min). The authors suggest that Alcalase hydrolysates are a favorable substrate for the hydrolysis by Flavourzyme, probably because Alcalase increases the amount of N-terminal sites in the protein and thus facilitates hydrolysis by the exoprotease activity of Flavourzyme. Both Alcalase and the sequential use of both enzymes achieved the degradation of proteins with a molecular weight higher than 15 kDa. However, the composition of the most prominent molecular weight peptide fraction in these hydrolysates (<15 kDa) was not reported in detail.

Another factor that is inherently related to the use of different enzymes is the pH of the hydrolysis process. However, the pH value of the hydrolysis can adversely influence the solubility of the protein which, in turn, directly affects its recovery from the hydrolysate. Bučko et al. [27] obtained a higher protein recovery from pumpkin* (Cucurbita pepo)* seed protein hydrolysates at pH 8 hydrolyzed by Alcalase (19.3 ± 0.6%) than at pH 3 hydrolyzed by Pepsin (15.9 ± 1.0%). The authors attributed this difference not only to the specific ability of the enzyme to break the peptide bonds but also to the pH of the medium. However, since the solubility of the protein is directly dependent on the pH value, the recovery of the protein can be enhanced posterior to the hydrolysis by adjusting the pH and the ionic strength of the solution.

The time of hydrolysis may also significantly influence the hydrolytic breakdown of Cucurbitaceae seed protein. Typically, the DH value increases with increasing hydrolysis time. Such is the case of the study performed by Siddeeg et al. [25] who prepared hydrolysates from seinat (*Cucumis melo* var.* tibish*) seed protein isolates, using a sequential treatment by Trypsin and Pepsin (both E/S ratios 1/100 [w/w]). First, the protein isolate solutions were incubated with Trypsin for 180 min at 37°C. After the inactivation of Trypsin by adjusting the pH value from 8 to 7, Pepsin was added to the samples. The hydrolysis was stopped at different times (30, 60, 90, 120, and 180 min), obtaining a steady kinetic curve with falling reaction rate over time. Thus, the DH values increased over time and ranged from 11.27 to 28.23%. These DH values, however, are relatively low in comparison to other proteases revised in this section. The authors hypothesize that, for this particular type of protein, alkaline proteases (such as Alcalase) might have higher activity compared to neutral and acid proteases (such as Trypsin and Pepsin, used in their experiment).

As for more complex effects of the time of hydrolysis, Popović et al. [10] observed an interaction between this factor and the type of enzyme used. The authors compared three different proteases (Alcalase, Flavourzyme, and Pepsin) in the hydrolysis of cucurbitin obtained from pumpkin* (Cucurbita pepo)* seed oil cake. The final degree of hydrolysis (*t* = 120 min) for Alcalase and Pepsin was very similar (27.1% and 29.0%, resp.) and much higher than for Flavourzyme (8.5%). The rate of Pepsin hydrolysis was constant, resulting in a steadily increasing degree of hydrolysis during the whole of 120 min. However, when Alcalase and Flavourzyme were used, the degree of hydrolysis increased rapidly in the first 30 min and remained constant for the rest of the treatment. Thus, the authors suggest that different time of hydrolysis can result in hydrolysates with different DH, depending on the type of enzyme used.

In order to detain the hydrolysis process of Cucurbitaceae seed protein, the enzymes are deactivated and the hydrolytic reaction stops. This is most commonly achieved by heating the reaction mixture up to 95–100°C, usually by submerging the reactor in boiling water for a short period of time (5–15 min). The mixture is then cooled down to room temperature and, sometimes, the pH value is adjusted in order to precipitate the indigested protein. The reaction mixture is then centrifuged and the supernatant is used for the determination of DH [30].

After the DH analysis, the supernatant is usually freeze-dried or spray-dried and the resulting protein hydrolysate powder is refrigerated (temperatures ranging from −18°C to 4°C). If NaCl is used in the hydrolysis, the supernatant may be dialyzed against water before drying [10]. The dried protein hydrolysate powder is then analyzed for an array of chemical and physical properties of interest, including the amino acid composition, the molecular weight distribution, and the bioactive and functional properties of the peptides.

## 3. Composition and Bioavailability of Cucurbitaceae Seed Protein Hydrolysates

Bioactive and functional properties of proteins, their isolates, and their hydrolysates are known to depend on their composition in terms of peptides and amino acids [28]. Crucially, the peptide bond hydrolysis improves the bioactivity of protein hydrolysates compared to their parent proteins, mainly due to the generation of bioactive peptides [31, 32]. Bioactive peptides can range in size from 2 to 20 or even 50 amino acid residues [41–44] but generally do not exceed 6–12 amino acid residues (<0.8–1.5 kDa, [29, 45]). The bioavailability of these peptides in target tissues depends directly on their molecular size, which greatly affects their absorption across the enterocytes [32]. Low-molecular weight peptides (bi- and tripeptides) can be absorbed intact and hydrolyzed later, whereas other oligopeptides (4–10 amino acid residues) and polypeptides (>10 amino acid residues) are usually hydrolyzed before being absorbed in the intestinal mucosa as free amino acids [41] which causes their bioactivity to drop dramatically [44]. Therefore, the intensity of peptide bioactivity is usually inversely correlated to the peptide length [44]. However, some of the larger peptides are able to resist degradation by digestive enzymes [41]. This depends on their amino acid composition and location within the sequence of amino acids that form the peptide [32, 34, 40].

Importantly, very few bioactive peptides from any vegetal and animal sources have been studied* in vivo* despite the years of extensive* in vitro* research. Thus, not much evidence exists in favor of bioactivity of these peptides in humans. As for* in vivo* studies of peptides from Cucurbitaceae seed protein hydrolysates in humans, none seem to exist at the moment. This is mainly due to the fact that Cucurbitaceae seed protein hydrolysates have yet to be thoroughly characterized in terms of their peptide composition and first studied* in vivo* in laboratory animals, such as rats.

Molecular weights of most abundant peptide fractions found in Cucurbitaceae seed protein hydrolysates by means of SDS-PAGE (sodium dodecyl sulphate-polyacrylamide gel electrophoresis) can be consulted in Table 1. The existing research on Cucurbitaceae seed protein hydrolysates as a potential source of bioactive peptides has mostly focused on bioactive and functional properties of the hydrolysates as a whole, consisting of several molecular weight fractions at once (Table 1). However, a few particular polypeptides and oligopeptides with different bioactive properties* in vitro* have already been identified in Cucurbitaceae seed proteins (Table 2). As for low-molecular weight peptide fractions (<1.5 kDa) which could potentially represent a source of bioactive and bioavailable bi- and tripeptides, these were also reported by many of the revised research papers (Table 1). However, such low-molecular weight peptides have yet to be adequately extracted and isolated from the peptide mixtures and subsequently studied in terms of their bioactivity, both* in vitro* and* in vivo* [44, 46].

The amino acid profile of protein isolates and hydrolysates obtained from seeds of several Cucurbitaceae species is shown in Tables 3 and 4, respectively. It is evident that the analyzed Cucurbitaceae seed protein isolates and hydrolysates contain a majority of essential and nonessential amino acids. Thus, Cucurbitaceae seed protein isolates and hydrolysates can be considered high-quality nutritional sources since they meet most nutritional requirements for body performance in terms of amino acids [17, 24]. Moreover, there is evidence for a direct link between several amino acids (Asp, Glu, Pro, Arg, His, Met, Leu, Ile, Ala, Tyr, and Val) and the antioxidant properties of protein hydrolysates [24, 47]. However, the absorption of amino acids in form of short peptide chains (di- and tripeptides) seems to be more efficient than the absorption of an equivalent amount of free amino acids, mainly due to the presence of peptide-specific transport systems and the subsequent peptide digestion into amino acids within the enterocytes [29].

## 4. Bioactive Properties of Cucurbitaceae Seed Protein Isolates and Hydrolysates

### 4.1. Antioxidant Activity

The determination of antioxidant activity in Cucurbitaceae seed protein usually comprises different* in vitro* measures, such as radical scavenging activity (for DPPH, ABTS^+^, and O_2_^−^ radicals), reducing power (of Fe^3+^ to Fe^2+^), and metal chelating activity (Fe^2+^), among others [48, 49].

Outstanding* in vitro* antioxidant activity has been reported for the globulin fractions (cucurbitin) of watermelon* (Citrullus lanatus)* [50] and pumpkin* (Cucurbita moschata)* [51] seed protein. Also, antioxidant effects of pumpkin* (Cucurbita pepo)* seed protein isolate have been reported* in vivo* in CCl_4_ induced liver injury in rats [52]. As for Cucurbitaceae seed protein hydrolysates, their antioxidant activity seems to be improved in comparison to unhydrolyzed seed protein [17]. However, it may depend on various hydrolysis-related factors, such as the type of enzyme used for the hydrolysis, the DH, and the molecular weight of the resulting peptides.

Vaštag et al. [22] studied the antioxidant activity of Alcalase and Pepsin hydrolysates of cucurbitin obtained from pumpkin (*Cucurbita pepo* L. c. v. “Olinka”) seed oil cake. The use of different enzymes for the hydrolysis did not significantly affect the ABTS radical cation scavenging activity (3.3–3.4 mmol/l TEAC for both Alcalase and Pepsin hydrolysates). However, the Alcalase hydrolysate proved to have almost twofold higher reducing power than the Pepsin hydrolysate (*A*_700 nm_ of about 0.25 and about 0.14, resp.). The authors hypothesize this was due to the Alcalase hydrolysate containing more peptides or amino acids able to donate electrons in order to react with free radicals and form more stable products in comparison to the Pepsin hydrolysate.

Similarly, Popović et al. [10] hydrolyzed cucurbitin extracted from pumpkin* (Cucurbita pepo)* oil cake with three different enzymes (Alcalase, Pepsin, and Flavourzyme) and investigated the antioxidant activity (ABTS radical cation scavenging activity and reducing power) of the cucurbitin hydrolysates. Measured by both antioxidant tests employed, the Alcalase and Pepsin hydrolysates showed good antioxidant activity while the Flavourzyme hydrolysates did not present any significant antioxidant activity. Interestingly, Alcalase and Pepsin hydrolysates were also reported to have the highest DH and the lowest molecular weight out of the three enzymatic hydrolysates studied (Table 1).

Vaštag et al. [26] observed the radical scavenging activity and the reducing power of both the Alcalase and Flavourzyme hydrolysate of protein isolate obtained from pumpkin (*Cucurbita pepo* L. c. v. “Olinka”) seed oil cake. As for the highest values of radical scavenging activity, the Alcalase hydrolysate (60 min, DH 53.23%) had significantly higher ABTS radical cation scavenging activity (7.59 ± 0.08 mM TEAC/mg) than both the Flavourzyme hydrolysate (60 min, DH 37.17%; 3.27 ± 0.19 mM TEAC/mg) and the sequential use of Alcalase and Flavourzyme (at 90 min, DH 60.94%; 4.75 ± 0.05 mM TEAC/mg and at 120 min, DH 69.29%; 4.71 ± 0.05 mM TEAC/mg). Moreover, the reducing power was not significantly affected by the sequential use of Alcalase and Flavourzyme when compared to the use of Alcalase only (the maximum values of *A*_700 nm_ oscillated between 0.33 ± 0.03 and 0.38 ± 0.04). The authors attributed the results to the specificity of the enzymes used for the hydrolysis, since each enzyme hydrolyzes proteins to result in a diverse composition of the hydrolysates, the Alcalase, and the sequential hydrolysate consisting of peptides with lower molecular weight than the Flavourzyme hydrolysate (Table 1). They also concluded that using Alcalase for the hydrolysis of pumpkin* (Cucurbita pepo)* seed protein might be the best option to improve the antioxidant activity of the cucurbitin hydrolysate.

Nourmohammadi et al. [24] hydrolyzed pumpkin oil cake (*Cucurbita pepo* var.* Styriaca*) protein isolate with Alcalase and Trypsin and used response surface methodology in order to suggest optimal treatment conditions in terms of DPPH radical scavenging activity. A sample hydrolyzed with 2% Alcalase at 50°C for 3.5 h (DH 28.0 ± 0.7, peptide size < 6.5 kDa) would achieve 90% DPPH radical scavenging activity whereas, for 1% Trypsin, 35°C and 5 h of treatment (DH 23.0 ± 0.5) were suggested as the best conditions, achieving 78% DPPH radical scavenging activity. Moreover, the Alcalase hydrolysate exhibited significantly better total antioxidant properties and metal chelating activity than the Trypsin hydrolysate (exact values not reported). Thus, the authors concluded that for this specific Cucurbitaceae seed protein isolate, Alcalase hydrolysis is the optimal treatment in order to achieve best antioxidant properties. They speculated that both the enzyme specificity and the hydrolysis conditions determine the size, type, and composition of free amino acids and peptides in the final hydrolysate which, in turn, directly affects its antioxidant properties. Moreover, they suggest that the lower the molecular size of the resulting peptides in the hydrolysate, the higher its antioxidant activity.

However, seed proteins from different Cucurbitaceae families seem to work best with different enzymes. For example, Arise et al. [23] hydrolyzed watermelon (*Citrullus lanatus* L.) seed protein isolates with Pepsin, Trypsin, and Alcalase and determined the antioxidant activity of the hydrolysates (reducing power and superoxide anion radical scavenging activity). The highest ferric-reducing ability was attained by the Trypsin hydrolysate (exact values not reported), whereas the Pepsin hydrolysate showed the highest O_2_^−^ radical scavenging activity (IC_50_ of 2.414 mg/mL, compared to 2.824 mg/mL for Trypsin and 3.205 mg/mL for Alcalase). Therefore, Alcalase does not seem to be the most suitable enzyme for the hydrolysis of this particular protein.

The antioxidant activity also seems to depend on the DH of the hydrolysate and on the molecular weight distribution of the peptides in the hydrolysate. In general, the higher the DH and the smaller the size of the peptides, the better the antioxidant activity of Cucurbitaceae seed protein hydrolysates. In Popović et al. [10], both the Pepsin and Alcalase hydrolysates had better ABTS radical cation scavenging activity and reducing power at higher DH values and lower molecular weights. Similarly, Vaštag et al. [26] observed that the radical scavenging activity and the reducing power of both the Alcalase and Flavourzyme hydrolysate were dependent on their DH and the size of the resulting peptides. However, while the radical scavenging activity of both hydrolysates increased with increasing DH, the reducing power decreased with increasing DH, except for the Alcalase hydrolysate whose reducing power increased with the DH but only up to DH of 40%.

Moreover, Siddeeg et al. [25] reported a similar trend when studying the effects of the hydrolysis time on the antioxidant activity of hydrolysates from seinat (*Cucumis melo* var.* tibish*) seed protein isolates, using a sequential treatment by Trypsin and Pepsin (both E/S ratios 1/100 [w/w]). At various hydrolysate concentrations (1–5 mg/mL), the authors observed that the DPPH radical scavenging activity, reducing power, ABTS radical scavenging activity, and Fe^2+^ chelating activity all gradually increased with increasing DH (11.27% at 30 min to 28.23% at 180 min). As for the best values, DPPH radical scavenging activity (5 mg/mL of hydrolysate) increased from 58.83 to 78.0% with the increase of DH, as well as the ABTS radical cation scavenging activity which significantly improved from IC_50_ of 3.09 mg/mL to 2.25 mg/mL. Also, the highest percentage of ferrous chelating activity (71.13%) was found at the highest DH (28.23%) at the concentration 2.0 mg/mL. Interestingly, molecular weight distribution of the hydrolysates did not change dramatically over the course of the hydrolysis (30–180 min), although the authors mention slight changes towards lower molecular weights.

Since none of the* in vitro* methods revised in this section measure antioxidant activity using living cells, it would be desirable to carry out experiments of such nature before studying the antioxidant activity of Cucurbitaceae seed protein hydrolysates* in vivo*.

### 4.2. Antihypertensive (ACE Inhibitory) Activity

Treating hypertension with synthetic drugs with angiotensin-I converting enzyme (ACE) inhibitory activity can have undesirable side effects, so food-derived peptides with ACE inhibitory activity are considered to be a better alternative [42, 53]. Food-derived peptides are encrypted in proteins and are usually released during food ripening or fermentation but can also be prepared by means of* in vitro* enzymatic hydrolysis [54]. While Cucurbitaceae seed protein isolates do not seem to have a significant ACE inhibitory activity [26], Cucurbitaceae seed protein hydrolysates could be a valuable source of peptides with ACE inhibitory activity [22, 26, 33].

The ACE inhibitory activity of peptides depends on various factors. As for the peptides derived from Cucurbitaceae seed proteins, their ACE inhibitory activity seems to directly depend on their concentration: the higher the concentration of the peptide, the higher its ACE inhibitory activity [33]. Moreover, the ACE inhibitory activity of Cucurbitaceae seed protein hydrolysates which contain the peptides seem to be dependent on the type of enzyme used for the hydrolysis, on the DH, and on their molecular weight distribution [22, 26]. However, the results are inconclusive and more research is needed in this area.

Priyanto et al. [33] identified peptides with ACE inhibitory activity in bitter melon* (Momordica charantia)* seed protein enzymatic (thermolysin) hydrolysates (Table 2). The hydrolysates were fractionated by HPLC and an ACE inhibitory assay was carried out on the different fractions. Crucially, a novel ACE inhibitory peptide (VY-7: VSGAGRY; molecular weight: 0.7 kDa) was found in momordin A proteic fraction (GI:157,832,029), having the best IC_50_ value of all identified peptides in the hydrolysate (8.64 ± 0.60 *μ*M).* In vivo* (studied in spontaneously hypertensive rats), the bitter melon* (Momordica charantia)* seed protein hydrolysate (at 10 mg/kg of body weight) showed moderate antihypertensive effects which became very pronounced for the VY-7 peptide alone (at 2 mg/kg of body weight).

As for the type of enzyme used for the hydrolysis, Vaštag et al. [22] hydrolyzed pumpkin (*Cucurbita pepo* L. c. v. “Olinka”) oil cake cucurbitin and found that both Alcalase and Pepsin hydrolysates proved to have ACE inhibitory activity (apparent IC_50_ values of 0.0244 mg and 0.0445 mg, resp.). However, Vaštag et al. [26] observed different ACE inhibitory activities for pumpkin oil cake (*Cucurbita pepo* L. c. v. “Olinka”) protein isolate hydrolysates by Alcalase, Flavourzyme, and the sequential use of both enzymes. While both the unhydrolyzed pumpkin seed protein isolate and the Flavourzyme hydrolysate showed no ACE inhibitory activity, for the Alcalase and the sequential hydrolysate, the ACE inhibitory activity depended on the DH. The highest ACE inhibitory activity (71.05 ± 7.50%; EC_50_ 0.442 mg/mL) was observed for the Alcalase hydrolysate with DH of 53.23 ± 0.70%. Below this DH, the ACE inhibitory activity of the Alcalase hydrolysate was lower. However, the sequential use of both enzymes (Alcalase and Flavourzyme) also showed a lower ACE inhibitory activity (about 55%), despite a higher DH (69.29 ± 0.90). The authors speculate that the ACE inhibitory peptides that were liberated when the Alcalase hydrolysate reached molecular weights below 15 kDa were probably broken down by the subsequent use of Flavourzyme which, in turn, caused the drop in the ACE inhibitory activity.

### 4.3. Antihyperglycaemic (*α*-Amylase Inhibitory) Activity

In the management of type 2 diabetes, *α*-amylase inhibitors can delay the absorption of glucose since *α*-amylase is responsible for the breakage of starch into products with low-molecular weight, such as glucose and maltose [55]. Therefore, substances with *α*-amylase inhibitory activity can be found in many commercially available antihyperglycaemic drugs. However, Cucurbitaceae seed protein isolates and hydrolysates may also be an interesting source of compounds with such properties [56]. In Cucurbitaceae seed protein isolates, *α*-amylase inhibitory activity has been reported for* in vivo* studies [57]. However, Cucurbitaceae seed protein isolates do not seem to possess *α*-amylase inhibitory activity* in vitro* and for Cucurbitaceae seed protein hydrolysates there are only a few* in vitro* studies of *α*-amylase inhibitory activity [22, 23].

Vaštag et al. [22] hydrolyzed pumpkin (*Cucurbita pepo* L. c. v. “Olinka”) oil cake cucurbitin and found that both Alcalase and Pepsin hydrolysates showed modest *α*-amylase inhibitory activity (<30%), the Pepsin hydrolysate showing slightly higher *α*-amylase inhibition than the Alcalase hydrolysate at all studied protein concentrations (0.5-2.0 mg/mL). However, Arise et al. [23] reported potent *α*-amylase inhibition (beyond 50%) for Pepsin, Trypsin, and Alcalase hydrolysates of watermelon (*Citrullus lanatus* L.) seed protein isolates at all studied protein concentrations (0.5–2.0 mg/mL). The Alcalase hydrolysate showed the strongest *α*-amylase inhibition, followed by the Trypsin and the Pepsin hydrolysate. The authors speculate that the strong *α*-amylase inhibition of Alcalase and Trypsin hydrolysates may be due to the enzymes creating specific (cationic and/or branched) amino acid residues. However, both Alcalase and Pepsin hydrolysates achieved a significantly lower IC_50_ in comparison to the Trypsin hydrolysate (0.149, 0.165, and 0.234 mg/mL, resp.).

As mentioned before, neither Vaštag et al. [22] nor Arise et al. [23] reported molecular weight distribution of peptides in the hydrolysates. This kind of information could be very valuable for linking the *α*-amylase inhibitory activity of the hydrolysate to peptides of particular size and structure.

## 5. Functional Properties of Cucurbitaceae Seed Protein Isolates and Hydrolysates

### 5.1. Protein Solubility

Solubility can influence other functional and bioactive properties of protein isolates and their hydrolysates and thus, it is considered one of the most important characteristics of protein [43, 58].

Protein solubility depends on the surface hydrophobic-hydrophilic balance of the protein and on the protein-protein and protein-solvent interactions [59]. These interactions are caused by charged, polar, and nonpolar groups of amino acid residues present on the surface of the protein [60]. Therefore, the solubility of Cucurbitaceae seed protein isolates and hydrolysates is highly dependent on the pH value of the solution. As a result of this, a pH solubility profile of Cucurbitaceae seed protein isolates and hydrolysates is often carried out by determining the protein solubility at different pH values. The Cucurbitaceae seed protein isolates and hydrolysates are dissolved in an aqueous solution, being occasionally stirred. After a certain amount of time (60 min), the mixture is centrifuged and the amount of protein that has dissolved is determined in the supernatant. The protein solubility is then expressed as a percentage of the dissolved protein to the total protein [w/w].

A typical pH solubility profile of Cucurbitaceae seed protein isolates is a U-shaped curve [10] on which the solubility of the protein reaches minimum values around its isoelectric point (pI). In Cucurbitaceae seed protein isolates, the pI usually corresponds to slightly acidic pH values (4-5) and a relatively low solubility of the protein isolate (0–15%). However, below pH 3 and above pH 5, the solubility of the protein isolates drastically improves, reaching the highest values at extreme pH values [18, 19, 61].

Wani et al. [19] carried out a pH solubility profile of watermelon* (Citrullus lanatus)* seed protein isolate (pH 1–12). The authors reported minimum solubility at the pH value of 4.0 (10.45–15.21%) and the highest solubility values (above 95%) were achieved at the pH values of 11 and 12. According to the authors, improved solubility of the watermelon seed protein isolate in the alkali region might be due to the higher amounts of aspartic and glutamic acid in the protein isolates. Similarly, Horax et al. [18] studied a pH solubility profile of bitter melon* (Momordica charantia)* seed protein isolate (pH 2–10). They observed the lowest protein solubility at pH values of 4.5–5.0 and the maximum protein solubility was reached at pH 2 (>80%) and in the alkali region of pH 7–10 (62.0–67.5%). The authors suggested that lower solubility of bitter melon* (Momordica charantia)* seed protein isolate in the alkali region may be due to a relatively low content of charged residues, such as aspartic or glutamic acid.

Additionally, Bučko et al. [61] investigated the effect of pH, ionic strength, and suspension concentration of pumpkin* (Cucurbita pepo)* seed protein isolate on its solubility. As for the influence of pH in the studied range of values (pH 3–8), the lowest solubility was observed at pH 5 (11% [w/v]) and the highest solubility at pH 8 (68% [w/v]). Furthermore, the influence of suspension concentration of protein isolate on its solubility was studied at different pH values (3, 5, and 8). Regardless of the suspension pH value, the authors observed an increase in the concentration of dissolved proteins with increasing suspension concentration. However, the solubility yield (the concentration of the dissolved protein to the suspension concentration) actually decreased with increasing suspension concentration but only at pH values of 3 and 8. At pH 5, suspension concentration had no effect on the solubility yield of the dissolved protein. As for the influence of the ionic strength on the solubility of the protein, slight salting-in effects (an increase in solubility) were observed at pH 5 and 8 while also moderate salting-out effects (a decrease in solubility) occurred at pH 3.

Importantly, enzymatic hydrolysis introduces a series of changes to the solubility of Cucurbitaceae seed protein isolates. During hydrolysis, the protein is degraded into smaller peptides and, in general, its solubility increases, which can be especially evident near the pI of the original protein [31]. However, this might not be the case at extreme pH values (especially above pH 9) where the solubility of the unhydrolyzed protein may maintain higher values than the solubility of the enzymatic hydrolysates [10].

The differences in solubility of the Cucurbitaceae seed protein isolates and hydrolysates can be attributed to the reduction of secondary structure of the original protein during the hydrolysis, which exposes ionizable amino and carboxyl groups and, in turn, increases the hydrophilicity of the peptides present in the hydrolysates [10, 27]. As a result of enzymatic hydrolysis, the overall influence of the pH on the solubility of the protein is attenuated and both the characteristic U-shaped pH profile and the pI can be lost for the hydrolysates [10, 27].

Bučko et al. [27] studied the influence of enzymatic (Alcalase and Pepsin) hydrolysis of pumpkin* (Cucurbita pepo)* seed protein isolate on protein solubility at different pH values, ionic strengths, and suspension concentrations. Both hydrolysates had the same DH (19%) and significantly higher solubility in comparison to the unhydrolyzed protein isolate, previously characterized in Bučko et al. [61]. This was particularly obvious near the isoelectric point of the pumpkin seed protein isolate (pI = 5) where the solubility of the protein isolate, the Alcalase hydrolysate, and the Pepsin hydrolysate was 12%, 68%, and >90%, respectively. Over the whole pH range (pH 3–8), the solubility of the Alcalase hydrolysate followed a slightly increasing trend (in the range of 60–76%). On the contrary, the solubility of the Pepsin hydrolysate decreased over the whole pH range but stayed above 90% at all pH values. The effects of the ionic strength on the protein solubility were also modulated by the hydrolysis. The Alcalase hydrolysate was the least influenced by ionic strength when compared to the protein isolate and the Pepsin hydrolysate. However, the authors claim that the overall effect of ionic strength seems to be a result of various factors, such as electrostatic interactions, ion specific effects, and hydrophobic effects, among others.

Similarly, Popović et al. [10] studied the pH profile of cucurbitin from pumpkin (*Cucurbita pepo* L. c. v. “Olinka”) oil cake and its enzymatic hydrolysates (Alcalase, Flavourzyme, and Pepsin). The solubility profile of cucurbitin was typically U-shaped, with high solubility at acidic and basic pH values and extremely low solubility in the pH range of 5–7 (0.05 mg/mL). Pepsin produced hydrolysates without a pI and with better solubility over the whole pH range, comparably to the results obtained by Bučko et al. [27]. However, Popović et al. [10] observed that Alcalase and Flavourzyme maintained the U-shaped pH solubility profile of the cucurbitin but reduced the pI to pH 5 (Alcalase) and pH 5-6 (Flavourzyme) in comparison to the unhydrolyzed protein (pH 5–7).

In addition to the aforementioned factors (pH, suspension concentration, and ionic strength), the solubility of the Cucurbitaceae seed protein hydrolysates also seems to depend on the DH, which in turn depends on the particular enzyme used for the hydrolysis. In general, the higher the DH of hydrolysis, the higher the solubility of the hydrolysate, as observed by Popović et al. [10]. The authors carried out the pH profile of cucurbitin and its enzymatic hydrolysates (Alcalase, Flavourzyme, and Pepsin) at different DH (14.6% and 29.8% for Alcalase, 5.6% and 9.2% for Flavourzyme, and 15% and 29% for Pepsin). They observed better solubility for the higher values of DH for all three enzymes in comparison to the lower values of DH and over the whole spectrum of pH values (2–11).

The solubility of protein hydrolysates may also directly depend on the time of hydrolysis. In theory, hydrolysis time should have the same effect as the DH, since longer hydrolysis times lead to higher DH. This was observed by Siddeeg et al. [25] who prepared Trypsin and Pepsin hydrolysates from seinat (*Cucumis melo* var.* tibish*) seed protein isolate at different times (30, 60, 90, 120, and 180 min). However, the authors also observed a decrease in solubility of the hydrolysates over hydrolysis time (instead of the expected increase). Moreover, they noted similar effects of the hydrolysis time on other functional properties, such as water and oil absorption capacities, emulsifying activity, and foaming capacity of the hydrolysates. This may point to possible adverse effects of long hydrolysis times on the quality of the final product. In fact, excessive hydrolysis of protein has been suggested to diminish the functional properties of the hydrolysate despite its increased solubility [28].

### 5.2. Emulsifying and Foaming Properties

Cucurbitaceae seed protein isolates and hydrolysates could be potentially used as functional food ingredients thanks to their balanced amino acid composition and their bioactive properties. However, the incorporation of protein isolates and hydrolysates into foods requires certain functional properties. In addition to enhanced solubility, it is crucial to find out whether Cucurbitaceae seed protein isolates and hydrolysates possess favorable emulsifying and foaming properties. These functional properties seem to be closely related to the size of the peptides conforming the hydrolysate, with excessively small peptides affecting adversely the functionality of the hydrolysate [25].

In order to investigate the emulsifying properties of Cucurbitaceae seed protein isolates and hydrolysates, emulsions of a vegetable oil (sunflower, soy, or corn, at 20–25%) with the isolates or hydrolysates are prepared at a concentration of dissolved protein, then homogenized, and analyzed. The analysis can include measuring the absorbance at 500 nm, where the absorbance at 0 min time is the emulsifying capacity (EC) and the decrease in absorbance in time is the emulsion stability (ES), or measuring of the diameter of the emulsion droplets. As for the foaming properties of Cucurbitaceae seed protein isolates and hydrolysates, air is introduced to the protein solution in phosphate buffer. The foam volume at 0 min represents the foaming capacity (FC) and the foam stability (FS) is expressed as the decrease in the foam volume over time.

Emulsifying and foaming properties of Cucurbitaceae seed protein isolates have been studied in bitter melon* (Momordica charantia)* seed protein isolate [18] and in watermelon* (Citrullus lanatus)* seed protein isolate [19]. In these Cucurbitaceae seed protein isolates, emulsifying and foaming properties are normally assessed at pH values of about 7 [18, 19]. This is because these functional properties depend on protein solubility which, in turn, is pH dependent. However, Bučko et al. [61] investigated emulsifying properties of pumpkin seed* (Cucurbita pepo)* protein isolate at different pH values (3, 5, and 8) and ionic strengths (0 and 0.5 mol/dm^3^ NaCl) of the emulsions. The emulsifying capacity and emulsion stability correlated with the results of protein solubility at the different pH values. This suggests that the principal limitation of Cucurbitaceae seed protein isolates is their low solubility at their pI and, thus, low functional properties at these pH values. Moreover, FC and FS have been reported to be relatively low in cucurbitin extracted from* Cucurbita maxima* pumpkin seed [62].

In comparison, functional properties of Cucurbitaceae seed protein hydrolysates seem to be improved over a range of pH values and ionic strengths, especially at the pI of the protein they are prepared from. Bučko et al. [27] investigated emulsifying properties of pumpkin* (Cucurbita pepo)* seed protein isolate and its hydrolysates (Alcalase and Pepsin) at different pH values (3, 5, and 8) and ionic strengths (0 and 0.5 mol/dm^3^ NaCl) of the emulsions. While pumpkin seed* (Cucurbita pepo)* protein isolate failed to form emulsions at pH 5 at both ionic strengths studied and at pH 3 and ionic strength of 0.5 mol/dm^3^ NaCl, both of its hydrolysates (Alcalase and Pepsin) showed improved emulsifying properties regardless of pH and ionic strength of the emulsion. These results suggest that enzymatic hydrolysis of Cucurbitaceae seed protein isolates can improve their functional properties over a wide range of pH values and ionic strength conditions. Importantly, Bučko et al. [27] reported the presence of relatively large peptides in all hydrolysates (14–20, 24, and 36 kDa) which could be responsible for the excellent emulsifying properties of the hydrolysates. On the other hand, small-sized peptides resulting from long hydrolysis times are less effective in creating stable emulsions than peptides with higher molecular size resulting from shorter hydrolysis times [31]. Such is the case of a study performed by Siddeeg et al. [25], who observed a decrease in both EC and FC of seinat (*Cucumis melo* var.* tibish*) seed protein hydrolysates over hydrolysis time (30, 60, 90, 120, and 180 min).

Another factor that influences the emulsifying and foaming properties of* Cucurbitaceae* protein hydrolysates is the DH. Popović et al. [10] reported an improved EC and ES for cucurbitin from pumpkin (*Cucurbita pepo* L. c. v. “Olinka”) oil cake and its enzymatic hydrolysates (Alcalase, Flavourzyme and Pepsin) at pH 7 for DH up to 15%. However, DH of 29-30% negatively affected these emulsifying properties. These findings are in line with the previously mentioned effects of peptide molecular size on the functional properties of the hydrolysates. Popović et al. [10] observed more peptides with higher molecular weight (33 and 22 kDa) in hydrolysates with DH up to 15%, whereas these were practically absent in hydrolysates with DH over 15%. On the other hand, the foaming properties of the protein hydrolysates were improved independently of the DH. It is therefore possible that foaming properties of* Cucurbitaceae* protein hydrolysates are not as sensitive to excessive hydrolysis as emulsifying properties are.

## 6. Future Trends and Conclusions

For millennia, Cucurbitaceae seeds have been widely used both as a protein-rich food ingredient and a nutraceutical agent by many indigenous cultures. However, relatively little is known about the bioactive components of the Cucurbitaceae seed proteins and their specific effects on human health. Technological advances have made it possible to extract different protein fractions from Cucurbitaceae seeds in order for them to be further analyzed. Enzymatic hydrolysis* in vitro* has been implemented to simulate the breakdown of these proteins during digestion. Nevertheless, to evaluate the real effect that Cucurbitaceae seed protein isolates and hydrolysates might have on living organisms, it is necessary to validate the findings of* in vitro* studies by means of* in vivo* studies, both in laboratory animals and in humans. However, only a few* in vivo* studies in rats have been carried out with Cucurbitaceae seed protein isolates [52, 57]. As for Cucurbitaceae seed protein hydrolysates, there is a need for* in vivo* studies since none seem to exist at the moment.

Not many bioactive properties of Cucurbitaceae seed protein hydrolysates have been investigated beyond their antioxidant activity and ACE/*α*-amylase inhibitory activities. For example, some proteins and peptides isolated directly from the seeds have shown anticarcinogenic, antifungal, and antimicrobial properties [35–38, 63] (Table 2). However, these properties have not yet been studied in the Cucurbitaceae seed protein hydrolysates. As for the functional properties of Cucurbitaceae seed protein isolates and hydrolysates, their solubility has been investigated thoroughly since all the other functional properties depend on it. However, information on their emulsifying and foaming properties is still very scarce. It would be particularly interesting to explore the direct relationship between the distribution of peptide molecular weight in the Cucurbitaceae seed protein hydrolysates and their bioactive and functional properties. The body of research reviewed in this paper seems to suggest that bioactive properties could be attributed to low-molecular weight peptides (<6.5–15 kDa) while enhanced functional properties could depend on the presence of larger peptides (>12–15 kDa) in the hydrolysates. However, there also seems to be only a small range of molecular weights that would guarantee the bioactive and functional properties of the hydrolysates. Therefore, future research should focus on purifying and characterizing the individual bioactive peptides within Cucurbitaceae seed protein hydrolysates. The obvious next step would be determining the bioavailability of these peptides, both in animals and in humans.

In order to benefit from oil industry byproducts, such as Cucurbitaceae seed oil cakes, finding cost-effective methods for Cucurbitaceae seed protein isolation and hydrolysis is a crucial step for the industrial implementation of such processes. Future research in this area should focus on applying hydrolysis pretreatments to Cucurbitaceae seed proteins and on the use of green technologies with the objective of reducing both the costs and the impact they may have on the environment. Moreover, the balanced amino acid composition and the bioactive properties of Cucurbitaceae seed protein isolates, hydrolysates, and peptides call for their use as functional food ingredients. However, studying such complex food systems is not the same as studying protein isolates, hydrolysates, or peptides on their own. Therefore, a lot of future research is needed in these areas.

## Figures and Tables

**Table 1:** Hydrolysis parameters of Cucurbitaceae seed protein and bioactive and functional properties of their hydrolysates.

Seed type	Substrate	Enzyme	Process parameters				Hydrolysis degree (%)	Molecular weight distribution (kDa)	Properties	Reference
			E/S ratio	pH	*T* (°C)	*t* (h)				
Watermelon (*Citrullus lanatus* L.)	Protein isolate	Pepsin	1/100	2.2	37	5	19.38	Not reported	Antioxidant and *α*-amylase inhibitory activity	Arise et al. [23]
		Trypsin		8	37	5	26.26			
		Alcalase		8	60	5	13.16			

Pumpkin (*Cucurbita pepo* L. c. v. “Olinka”)	Protein isolate	Alcalase	1/757	8	50	1	53.23	<15	Antioxidant and ACE-inhibitory activity	Vaštag et al. [26]
		Flavourzyme	1/385			1	37.17	36–40 and <15		
		Sequential use (Alcalase + Flavourzyme)	A (1/250)			1	69.29	<15		
			F (1/385)			2				

Pumpkin (*Cucurbita pepo* L. c. v. “Olinka”)	Cucurbitin	Alcalase	2/100	8	50	1	26.94	Not reported	Antioxidant, ACE, and *α*-amylase inhibitory activity	Vaštag et al. [22]
		Pepsin		3	37		18.7			

Pumpkin (*Cucurbita pepo* L. c. v. “Olinka”)	Cucurbitin	Alcalase	2/100	8	50	0 to 2.5	29.8	22 and <12	Antioxidant activity, emulsifying, and foaming capacity	Popović et al. [10]
		Flavourzyme		7	50		9.2	33, 22, and <12		
		Pepsin		3	37		29	<12		

Pumpkin *(Cucurbita pepo)*	Protein isolate	Alcalase	0.5 ml/g	8	50	0.5	19	36, 24, and 20–14	Emulsifying capacity	Bučko et al. [27]
		Pepsin	2/100	3	37	1.5	19	20–14		

Pumpkin (*Cucurbita pepo con.Pepo* var. *styriaca*)	Protein isolate	Alcalase	1/100 to 2/100	9	45 to 55	2 to 5	28	<6.5	Antioxidant activity	Nourmohammadi et al. [24]
		Trypsin		8	35 to 45		23	Not reported		

Pumpkin *(Cucurbita moschata)*	Protein meal	Alcalase	1/100	8	55	5	13.84	1–0.18, 5–1, and <0.18^*∗*^	Antioxidant activity	Venuste et al. [17]
		Flavourzyme		7	50		11.8	>10, 1–0.18, and 5–1^*∗*^		
		Protamex		6.5			8.74	1–0.18, 5–1, and <0.18^*∗*^		
		Neutrase		7			4.12	5–1, 1–0.18, and <0.18^*∗*^		

Bitter melon *(Momordica charantia)*	Protein pellet	Trypsin	1/50	8	37	12	Not reported	2.5–0.7	ACE-inhibitory activity	Priyanto et al. [33]
		*α*-Chymotrypsin			37					
		Alcalase			50					
		Pepsin		1.5	37					
		Thermolysin	1/50 to 1/800	8	40 to 70	1 to 15				

Seinat (*Cucumis melo* var. *tibish*)	Protein isolate	Sequential use (Trypsin + Pepsin)	1/100	8	37	3	28.23	0.5–0.18, 1–0.5, and 1-2^*∗*^	Antioxidant activity	Siddeeg et al. [25]
				7	37	0.5 to 3				

Seinat (*Cucumis melo* var. *tibish*)	Protein fractions	Sequential use (Pepsin + Trypsin)	1/100	2 7	37 37	3 3	Not reported	Glutelin: 0.5–0.32 and 1–0.5^*∗*^; albumin 0.5–0.18 and 1–0.5^*∗*^; globulin: 1–0.5 and 0.5–0.18^*∗*^; glutelin: 0.5–0.18	Antioxidant activity	Siddeeg et al. [34]

^*∗*^Predominant molecular weight fractions in descending order.

**Table 2:** Polypeptides and oligopeptides with different bioactive properties *in vitro* identified in Cucurbitaceae seed proteins.

Source	Sequence	Molecular weight (kDa)	Properties	Reference
Pumpkin *(Cucurbita maxima)*	KRDPDWRREQEERREQERRREQQQQRREEQQRGER	12.33	Antifungal	Vassiliou et al. [35]
	YTRGGRGGWKGGGGGKGGGGGKGGGGG	2.34		

Pumpkin (*Cucurbita moschata* cv. black pumpkin)	PQRGEGGRAGNLLREEQEI	~9	Antifungal	Wang and Ng [36]

*Momordica cochinchinensis*	GCEGKQCGLFRSCGGGCRCWPTVTPGVGICSSS	3.29	Anticarcinogenic	Chan et al. [37]
	GCEGKPCGLFRSCGGGCRCWPTVTPGVGICSS	3.17		

*Benincasa hispida*	SDYLNNNPLFPRYDIGNVELSTAYRSFANQKAPGRLNQNWALTADYTYR	5.75	Antimicrobial and antioxidant	Sharma et al. [38]

Bitter melon *(Momordica charantia)*	FREKVYNIPL	1.28	ACE-inhibitory activity	Priyanto et al. [33]
	VSGAGRY	0.71		
	ITLPYSGNYER	1.31		
	IAAGKPREKIP	1.18		
	IAAGKPREKIPIGLPA	1.63		
	LLHYDSTAAAGALLVLIQTTAEAAR	2.57		
	LHYDSTAAAGALLVLIQTTAEAAR	2.46		
	LAQGNNGIFRTPIVL	1.61		
	IFRTPIVL	0.96		
	VDNKGNR	0.80		
	FFKESPPEA	1.05		
	FFKESPPEAYN	1.33		
	IISLENQWSA	1.16		
	FRNPVDL	0.86		
	QASESLN	0.75		
	LRYDDGWM	1.05		
	IVLSSATDKGNGQQWT	1.70		

**Table 3:** Amino acid composition (g/100 g) of Cucurbitaceae seed protein isolates.

Amino acid		Seinat (*Cucumis melo* var. *tibish*)	Bitter melon *(Momordica charantia)*	Watermelon *(Citrullus vulgaris)*	Watermelon *(Citrullus vulgaris)* cv. Mateera	Watermelon *(Citrullus vulgaris)* cv. Sugar baby	Pumpkin (*Cucurbita pepo con. Pepo* var. *styriaca*)
		Siddeeg et al. [25]	Horax et al. [18]	Jyothi lakshmi and Kaul [39]	Wani et al. [12]	Wani et al. [12]	Nourmohammadi et al. [24]
Essential amino acid	Histidine (His)	2.21	3.71	2.46	1.86	2.15	1.83
	Threonine (Thr)	2.87	1.6	3.47	3.49	3.17	1.88
	Valine (Val)	4.42	—	2.91	—	—	3.35
	Methionine (Met)	2.47	2.75	0.33	0.97	0.88	—
	Phenylalanine (Phe)	5.30	4.11	5.18	5.36	5.83	4.22
	Isoleucine (Ile)	3.40	3.27	1.44	5.17	5.21	2.88
	Leucine (Leu)	6.27	6.57	7.44	7.09	7.19	5.33
	Lysine (Lys)	2.76	8.49	2.18	3.21	2.92	2.65
	Tryptophan (Try)	—	—	1.07	0.96	1.17	—

Nonessential amino acid	Tyrosine (Tyr)	2.86	4.51	2.85	3.89	3.96	2.32
	Cysteine (Cys)	5.91	1.27	0.96	6.31	6.09	—
	Aspartic acid (Asp)	6.72	8.54	9.48	9.14	10.39	7.22
	Glutamic acid (Glu)	14.48	14.8	20.71	17.69	16.75	14.28
	Serine (Ser)	3.93	4.17	5.91	4.87	4.96	3.97
	Glycine (Gly)	3.86	4.13	5.82	4.86	5.04	4.23
	Arginine (Arg)	12.42	10.1	16.89	14.53	15.21	10.65
	Proline (Pro)	—	4.56	—	4.21	3.95	—
	Alanine (Al)	—	4.17	—	4.89	5.05	3.14

**Table 4:** Amino acid composition (g/100 g) of Cucurbitaceae seed protein hydrolysates.

Amino acid		Pumpkin *(Cucurbita moschata)*, Alcalase	Pumpkin *(Cucurbita moschata)*, Flavourzyme	Pumpkin *(Cucurbita moschata)*, Protamex	Pumpkin *(Cucurbita moschata)*, Neutrase	Pumpkin (*Cucurbita pepo con. Pepo* var. *styriaca*), Alcalase	Seinat (*Cucumis melo* var. *tibish*), Trypsin + Pepsin
		Venuste et al. [17]	Venuste et al. [17]	Venuste et al. [17]	Venuste et al. [17]	Nourmohammadi et al. [24]	Siddeeg et al. [25]
Essential amino acid	Histidine (His)	1.37	1.43	1.17	1.6	1.67	2.06
	Threonine (Thr)	1.88	1.59	1.74	1.77	2.01	2.64
	Valine (Val)	3.09	2.55	2.55	3.57	3.45	3.63
	Methionine (Met)	2.34^x^	1.99^x^	1.76^x^	2.38^x^	—	1.96
	Phenylalanine (Phe)	6.02^y^	4.61^y^	4.82^y^	6.2^y^	4.29	4.39
	Isoleucine (Ile)	2.72	3.00	2.57	2.73	3.10	3.36
	Leucine (Leu)	4.7	3.7	4.43	4.87	5.64	5.32
	Lysine (Lys)	2.44	2.2	2.58	2.49	3.00	1.78
	Tryptophan (Try)	0.52	0.77	0.15	0.16	—	—

Nonessential amino acid	Tyrosine (Tyr)	—	—	—	—	2.88	2.25
	Cysteine (Cys)	—	—	—	—	—	7.40
	Aspartic acid (Asp)	6.47	5.47	5.72	6.44	7.56	6.79
	Glutamic acid (Glu)	15.89	15.09	16.18	16.87	17.19	12.00
	Serine (Ser)	3.74	3.39	3.72	3.79	4.04	2.98
	Glycine (Gly)	3.86	4.28	3.92	4.06	3.95	4.08
	Arginine (Arg)	11.6	11.82	11.78	13.65	—	9.68
	Proline (Pro)	2.04	1.84	1.83	2.33	13.60	—
	Alanine (Al)	3.41	2.70	2.81	3.11	3.47	—

x: methionine + cysteine; y: tyrosine + phenylalanine.
